# Uni-axial stretch induces actin stress fiber reorganization and activates c-Jun NH_2_ terminal kinase via RhoA and Rho kinase in human bladder smooth muscle cells

**DOI:** 10.1186/s12894-016-0127-9

**Published:** 2016-02-29

**Authors:** Nobuhiro Kushida, Osamu Yamaguchi, Yohei Kawashima, Hidenori Akaihata, Junya Hata, Kei Ishibashi, Ken Aikawa, Yoshiyuki Kojima

**Affiliations:** Department of Urology, Fukushima Medical University School of Medicine, 1, Hikarigaoka, Fukushima, 960-1295 Japan; Division of Bioengineering and LUTD Research, Nihon University School of Engineering, Nihon University, 1, Nakagawara, Tokusada, Tamura, Koriyama, 963-8642 Japan

**Keywords:** Bladder smooth muscle, C-Jun NH_2_ terminal kinase, Mechanical stretch, RhoA, Rho kinase

## Abstract

**Background:**

Excessive mechanical overload may be involved in bladder wall remodelling. Since the activity of Rho kinase is known to be upregulated in the obstructed bladder, we investigate the roles of the RhoA/Rho kinase pathway in mechanical overloaded bladder smooth muscle cells.

**Methods:**

Human bladder smooth muscle cells were stimulated on silicon culture plates by 15 % elongated uni-axial cyclic stretch at 1 Hz. The activity of c-Jun NH_2_-terminal kinase was measured by western blotting and actin stress fibers were observed by stained with phallotoxin conjugated with Alexa-Fluor 594.

**Results:**

The activity of c-Jun NH_2_-terminal kinase 1 peaked at 30 min (4.7-fold increase vs. before stretch) and this activity was partially abrogated by the RhoA inhibitor, C3 exoenzoyme or by the Rho kinase inhibitor, Y-27632. Stretch induced the strong formation of actin stress fibers and these fibers re-orientated in a direction that was perpendicular to the stretch direction. The average angle of the fibers from the perpendicular to the direction of stretch was significantly different between before, and 4 h after, stretch. Actin stress fibers reorganization was also suppressed by the C3 exoenzyme or Y-27632.

**Conclusions:**

Bladder smooth muscle cells appear to have elaborate mechanisms for sensing mechanical stress and for adapting to mechanical stress overload by cytoskeletal remodeling and by activating cell growth signals such as c-Jun NH_2_-terminal kinase via RhoA/Rho kinase pathways.

## Background

Bladder wall remodeling such as smooth muscle hypertrophy/hyperplasia occurs under conditions of bladder outlet obstruction including benign prostate hyperplasia. Although these etiologies are not well understood, an excessive mechanical overload might be a prior factor. In the human obstructed bladder, sustained stretch stress is believed to cause bladder wall remodeling such as a change in the ratio of extracellular matrix and smooth muscle cells [1, 2]. In vivo animal models with a partial urethral ligature have revealed smooth muscle hypertrophy in the bladder wall, which are quite similar to the obstructed bladder in humans [3]. Although *in vitro* devices do not completely mimic the bladder wall overload, stretch devices that enable the stimulation of cultured bladder smooth muscle cells were used to reveal the pathological mechanisms under condition of mechanical overload. Park et al. indicated that angiotensin release induced by mechanical stretch acts as mitogen in bladder smooth muscle cells [4, 5].

Some studies have identified intracellular signaling pathways that mediate the biological effects evoked by mechanical stimuli and ultimately lead to nuclear events [5]. Of these pathways, the mitogen activated protein kinases (MAPKs), which constitute a family of serine/threonine kinases, are known to mediate signals that are activated by external stimuli and that regulate cell growth and differentiation. One MAPK family member, c-Jun NH_2_-terminal kinase (JNK), has been reported to be activated by mechanical stretch in vascular smooth muscle cells [6] and cardiac myocytes [7]. In addition, Nguyen et al. indicated that cyclic stretch activates JNK in bladder smooth muscle cells [8]. We also showed that stretch stimulation activated JNK in rat bladder smooth muscle cells by the influx of Ca^2+^ through a stretch activated ion channel [9].

RhoA is a member of the Rho family of 20 to 30 kDa GTPase proteins that cycle between an active GTP-bound form and an inactive GDP-bound form. One of the important roles of RhoA is to act as a regulator of actin stress fibers [10]. RhoA is involved in cell division, movement, polarization and morphological changes via reorganization of actin stress fibers. The Rho-associated coiled-coil forming protein kinase (ROCK) is a molecule of RhoA that acts as a serine/threonine kinase and phosphorylates various substrates. Actin stress fiber reorganization was recently reported to be mediated by the ROCK pathway [11]. The physical deformation of cells appears to be caused by reorganization of actin stress fibers in order to adapt to their extracellular environments.

Previous evidence suggested that the RhoA/ROCK pathway is involved in the pathogenesis of obstructed bladder [12] and in the change in Ca^2+^ sensitization due to agonist stimulation [13]. Poley et al. indicated that quick stretch of rabbit bladder smooth muscle sufficient to induce calcium entry and stimulate a myogenic contraction does not activate the ROCK, and that basally active ROCK is necessary for stretch induced myogenic contraction [14]. We undertook to identify the roles of the RhoA/ROCK pathway in the early signalling events evoked by mechanical stimuli in human bladder smooth muscle cells (HBSMCs).

## Methods

### Cultured HBSMCs

Commercially established HBSMCs (Cambrex Bio Science, Walkersville, USA) were used for all experiments. Cultured cells were identified by immunostaining with anti-α smooth muscle actin (Sigma, Saint Louis, USA). Cells were maintained in the growth medium: SmBM-2 with BulletKit containing 5 % fetal bovine serum (Cambrex) in a humidified 5 % CO_2_-95 % air atmosphere at 37 °C. All experiments were performed on cells between passages 2 and 4.

### Application of uni-axial mechanical cyclic stretch

HBSMCs were seeded on 35-mm square silicon elastomer bottomed culture plates that had been coated with 1 μg/ml fibronectin (Wako, Osaka, Japan) dissolved in phosphate buffer saline (PBS). After achieving 90 % confluency, the cells were subjected to uni-axial cyclic stretch using a controlled motor unit; ST-140 (Strex, Kyoto, Japan). The intensity of stretch was 15 % elongation and the stretch cycle frequency was 1 Hz. These procedures were carried out in a humidified incubator with 5 % CO_2_-95 % air at 37 °C.

### Protein extraction and Western blotting

Stimulated HBSMCs were harvested with a cell scraper and were solubilized in a lysis buffer consisting of 20 mM Tris–HCl (pH 7.5), 1 % Nonidet P-40, 1 mM EDTA, 50 mM NaF, 50 mM sodium β-glycerophosphate, 0.05 mM Na_3_VO_4_, 10 μg/ml leupeptin, and 100 μM phenylmethylsulfonyl fluoride. Following centrifugation at 5000*g* for 5 min, the resultant supernatant was used as the lysate after protein concentration determination using the Bradford assay (Bio-Rad, Hercules, USA). The lysates were resolved in a 10 % SDS polyacrylamide gel and were electrotransferred to Hybond-P Polyvinylidene difluoride membrane (GE healthcare, Amersham Place, UK). Immunodetection of JNK1 and phosphorylated JNK1 was performed using anti-JNK1 (1:1000 dilution; Cell Signaling, Massachusetts, USA), and anti-phosphorylated JNK1 (Thr183/Tyr185) antibodies (1:1000; Cell Signaling), respectively. The signal was detected by incubation with an anti-rabbit secondary antibody conjugated to horseradish peroxidase (1:10,000; Promega, Tokyo, Japan), followed by chemiluminescence detection using the SuperSignal kit (Pierce Chemical Company, Rockford, USA) and Kodak BioMax light film (Kodak, Tokyo, Japan). The densities of the bands were quantified using the computer software, ImageJ (developed at the U.S. National Institutes of Health).

### Labeling of actin stress fibers and nuclei

Following stretch stimulation, the cells were fixed in 3.7 % formaldehyde solution for 10 min and were permeabilized in a 0.1 % Triton X-100 solution for 5 min at room temperature. After incubation with PBS containing 1 % BSA for 30 min, the cells were stained with phallotoxin conjugated with Alexa-Fluor 594 (Molecular Probes, Eugene, USA) at 1:200 dilution for 20 min. Subsequently the cell nuclei were stained using 300 nM DAPI (Molecular Probes).

### Measurement of actin stress fiber angles

Actin-stained cell images were captured and digitized using the Olympus IX71 fluorescence microscopy system (Olympus, Tokyo Japan). The angles of formed actin stress fibers were measured by using ImageJ. Stretch fibers that were perpendicular to the stretch direction were defined as having an angle of 0° and stress fibers that were parallel to the stretch direction were defined as having an angle of 90°.

### Other chemicals

The *Clostridium botulinum* C3 exoenzyme and SP600125 were purchased from Sigma (Saint Louis, USA). Lipofectamine was from Invitrogen (Paisley, UK). Y-27632 was from Tocris Cookson Ltd (Bristol, UK).

### Statistics

Statistical analysis between groups was performed using the Kruskal-Wallis test and Dunn’s multiple comparison test for post-hoc comparison using the GraphPad Prism 6 software (GraphPad software, San Diego, USA). The null hypothesis was rejected at *p* < 0.05.

## Results

### The activation of JNK1 in HBSMCs exposed to uni-axial mechanical stretch

**Fig. 1:**
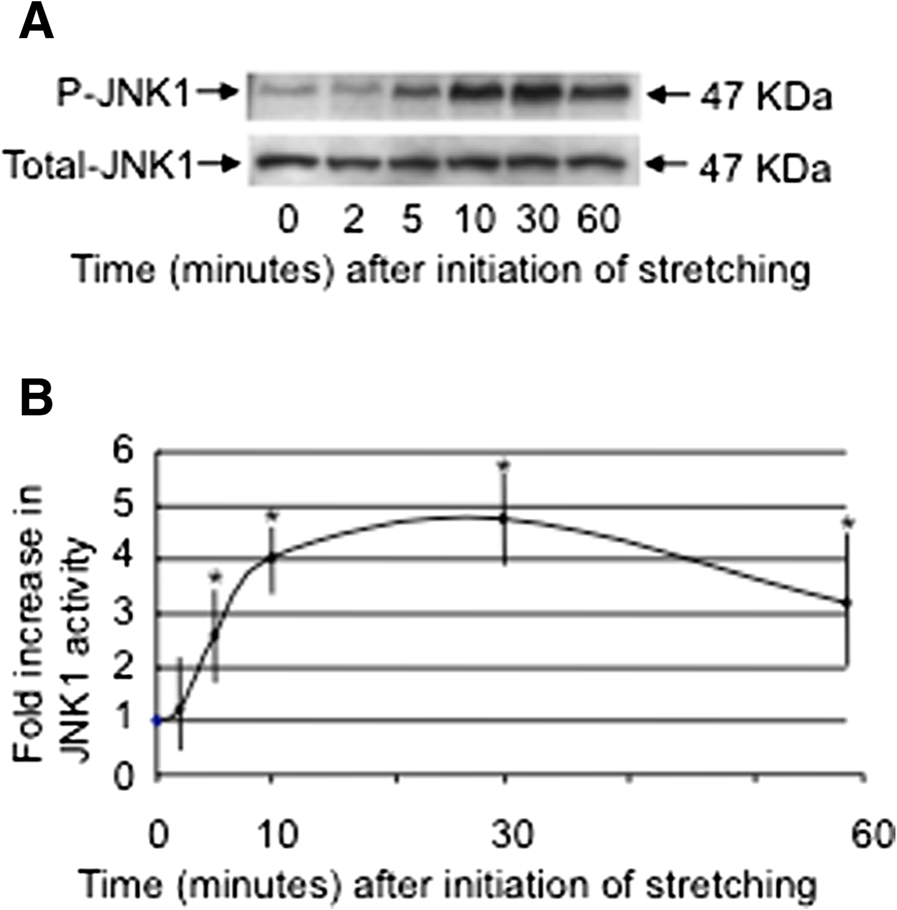
Time-dependent JNK1 activation by uni-axial cyclic mechanical stretching of HBSMCs. **a** HBSMCs were exposed to uni-axial stretch (15 % elongation) for the indicated periods. The activity of JNK1 was measured by Western blotting using an anti-phosphorylated JNK1 (Thr183/Tyr185) antibody, which recognizes the active form of JNK1 (phosphorylated JNK1; P-JNK). The same filter was immunoblotted with each of the specific antibodies to demonstrate the total amount of JNK1 (Total-JNK1). **b** Quantification of the activity of JNK1 at each time point after stretch application. JNK1 activity was quantified using densitometry. The results are shown as means ± SEM (*n* = 6). The data are normalized by the total protein amount, and the intensity at 0 min was set at 1.0. An * indicates *p* < 0.05 compared to the value at time 0

We first measured the activation of JNK1 following exposure of HBSMCs to 15 % elongation, 1 Hz cyclical uni-axial mechanical stretch over a period of 60 min. The activity of JNK1 was measured at each time point. The activity of JNK1 was enhanced from 5 min after stretching and peaked at 30 min, at which time a 4.7-fold increase in activation was detected (Fig. 1).

### Dependency of JNK1 activation on the RhoA/ROCK pathway

**Fig. 2:**
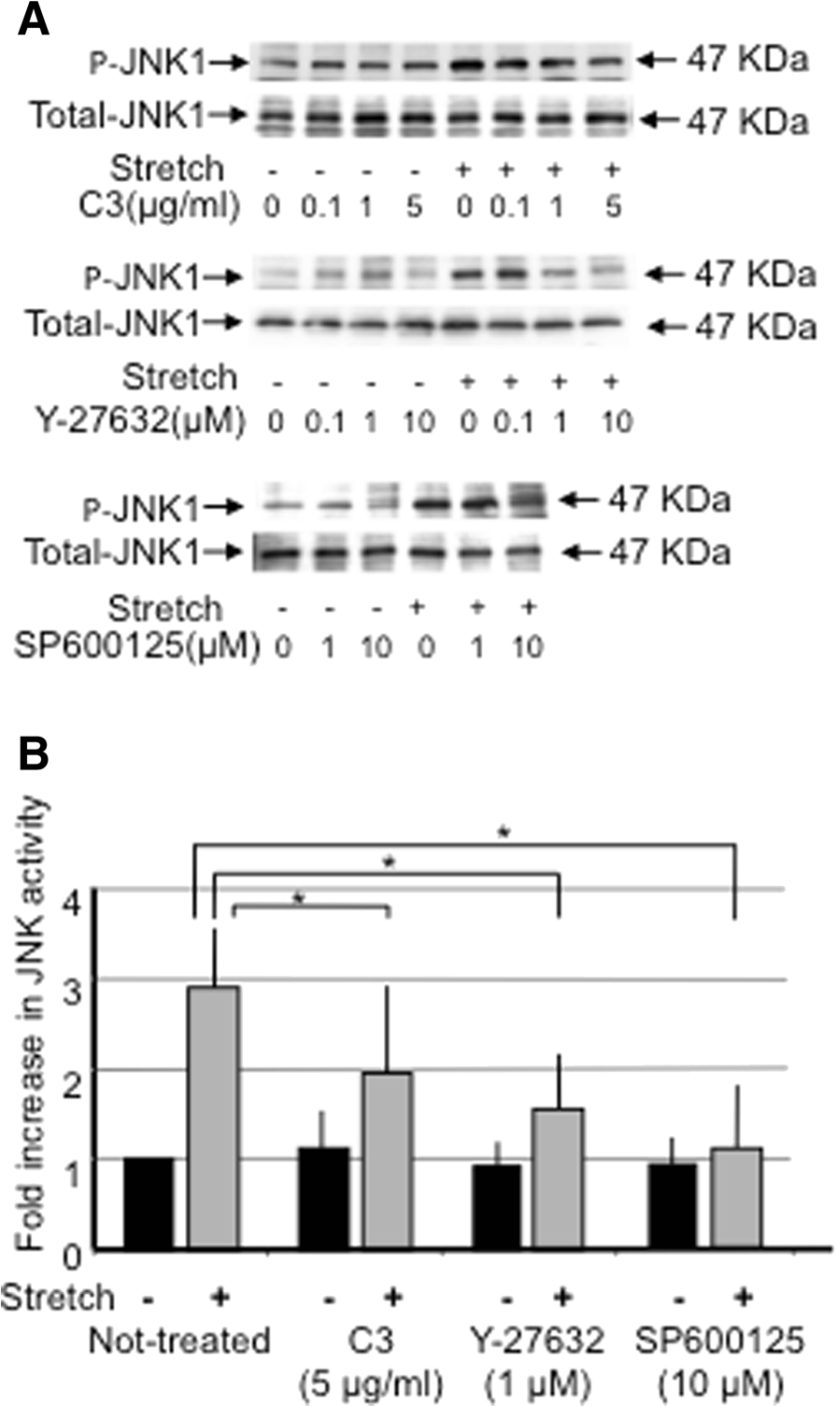
The effect of signaling inhibitors on stretch-induced JNK1 activation. **a** HBSMCs were pre-incubated with 10 μg/ml lipofectamine and the RhoA inhibitor C3 exoenzyme (upper blots), the Rho kinase inhibitor Y-27632 (middle blots), or the JNK1 inhibitor SP600125 (bottom blots) at the indicated concentrations and the cells were then subjected (+), or not (−), to uni-axial stretch with 15 % elongation for 30 min. The activity of JNK1 was measured in the same manner as in Fig. 1. **b** Quantification of the effect of the inhibitors on JNK1 activation. Stretch induced JNK1 activity was quantified by densitometry. JNK1 activity under the conditions of pre-incubation with 5 μg/ml C3 exoenzyme, 1 μM Y-27632 or 10 μM SP600125 are indicated in the figure. Data were normalized by the total amount of JNK1 protein and the intensity of non-stretched, non-treated cells was defined as 1.0. Data are indicated as means ± SEM (*n* = 6). An * indicates *p* < 0.05

Based on this result, 15 % elongation and a 1 Hz cyclic stretch for 30 min were employed as the stretch conditions for analysis of the role of the RhoA/ROCK pathway in JNK1 activation by uni-axial stretch. We first analyzed the effect of inhibition of RhoA using the *botulinum* C3 exoenzyme, which is a RhoA inhibitor that specifically ADP-ribosylates RhoA at asparagine 41. As described in a previous paper [15], HBSMCs were pre-incubated with 10 μg/ml lipofectamine in order to increase the permeability of the cell membrane before C3 exoenzyme treatment. Pre-incubation of the HBSMCs with the C3 exoenzyme for 30 min before stretch inhibited JNK1 activation by uni-axial stretch in a C3 dose-dependent manner (Fig. 2a-upper blot). In a similar manner, uni-axial stretch-induced JNK1 activation was also suppressed by 30 min pre-incubation with the ROCK inhibitor, Y-27632, in a dose dependent manner (Fig. 2a-middle blot). The JNK specific inhibitor; SP600125 also inhibited JNK1 activation (Fig. 2a-lower blot). JNK1 activity was decreased to approximately 66.2, 55.9 and 39.0 % of that of the control activity by C3, Y-27632 and SP600125, respectively (Fig. 2b). As RhoA and ROCK inhibitors suppressed JNK1 phosphorylation, it is possible that RhoA/ROCK signaling may have some effect on JNK1 activation evoked by uni-axial stretch.

### Actin stress fiber reorganization by uni-axial stretch

**Fig. 3:**
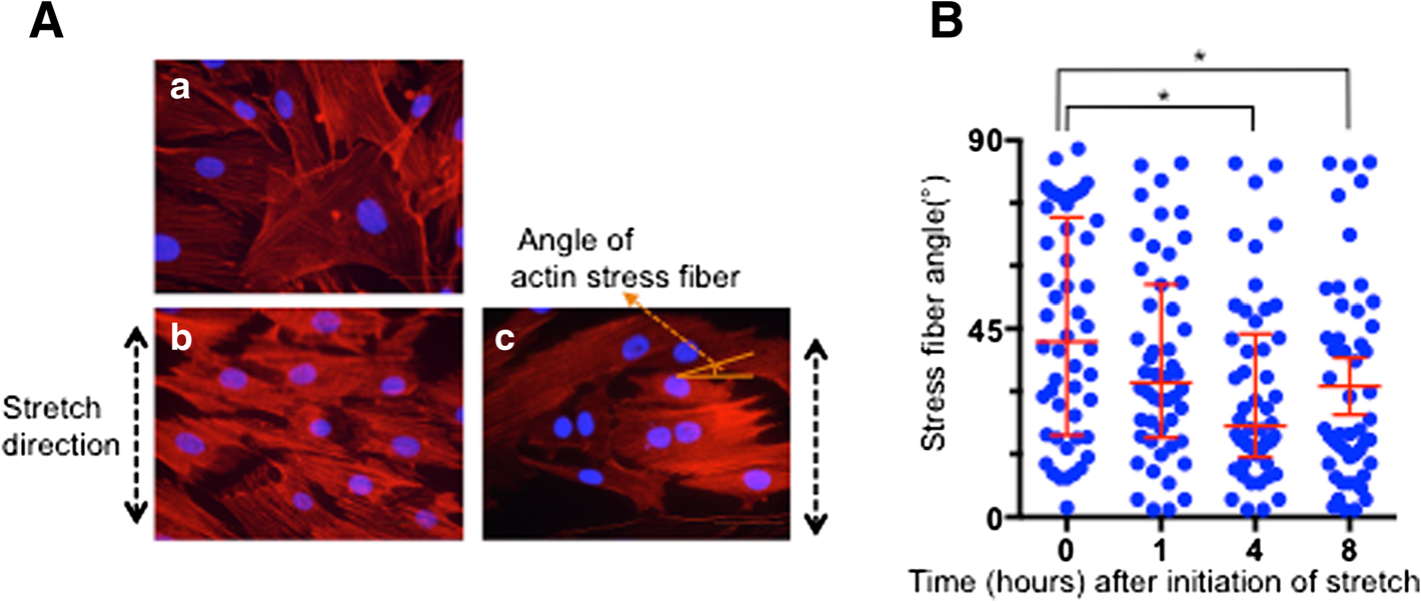
Actin stress fiber re-organization induced by mechanical stretch. **a** HBSMCs were exposed to uni-axial stretch (15 % elongation) for 1, 4 or 8 h and cellular actin was then stained with phalloidin conjugated with Alexa-Fluor 594 at 1:200 dilution (red). Nuclei were dyed blue using 300 nM DAPI. Representative staining of cells (*a*) before stretch and (*b, c*) after stretching for 4 h. (*c*) The scheme of the measurement of stretch fiber angles; Actin fibers at right angles to the stretch direction were defined as having an angle of 0° and those parallel with the stretch direction were defined as having an angle of 90°. The scale bar in pictures indicates 20 μm. **b** Quantification of the angles of actin stress fibers at each time point. The direction of the actin stress fibers in each cell was determined by averaging the angles of 5 obvious fibers within a cell (*n* = 50 cells per time point). Data are expressed as the median ± interquartile. An * indicates *p* < 0.05

We next determined the effect of exposure of HBSMCs to uni-axial stretch with 15 % elongation on the organization of actin stress fibers by staining of cellular actin with phalloidin. Compared with non-stretched cells, cells that had been stretched for 4 h showed stronger staining of actin stress fibers. The fibers had also re-orientated so as to be more perpendicular to the stretch direction. Typical pictures of phalloidin stained cells that were not exposed, or were exposed to uni-axial stretch are shown in Fig. 3a (*a* and *b*, respectively). The angle of the stress fibers in relation to the direction of stretch was measured. For this measurement, stress fibers that were perpendicular to the direction of the stretch were defined as having an angle of 0° and fibers that were parallel to the stress fibers were defined as having an angle of 90°. The measurement of these angles is shown in Fig. 3a*c*. The angle of the actin fibers in each cell was determined by taking the average value of 5 distinct fibers, and 50 cells were counted for each time point (Fig. 3b). The graph of these values indicated that the actin stress fibers in the cells moved in a direction that was perpendicular to the direction of stretch. The average angle of the actin stress fiber direction after 4 h of stretch was statistically different compared with that of non-stretched cells (41.9° [19.5, 71.6] before stretch vs. 21.7° [14.3, 43.8] [16] after 4 h of stretch. Median and interquartile values respectively are indicated in square brackets.).

### Inhibitor effects on stress fiber reorganization

**Fig. 4:**
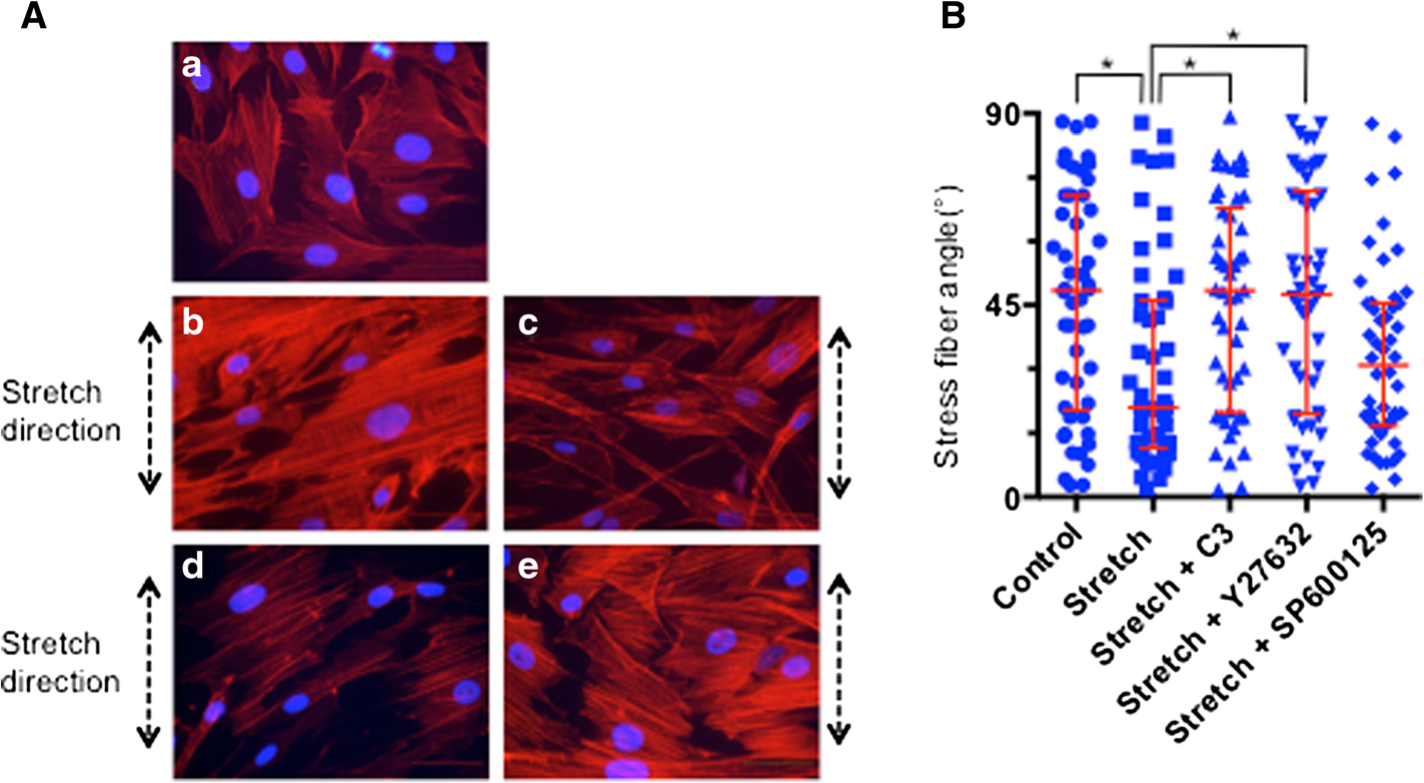
The effect of signal inhibitors on stress fiber re-organization. **a** HBSMCs were not treated with inhibitor and (*a*) not-stretched or (*b*) stretched for 4 h, or were pre-incubated with 10 μM lipofectamine and 5 μg/ml of the C3 exoenzyme (*c*), 1 μM of Y-27632 (*d*) or 10 μM SP600125 (*e*) for 30 min followed by uni-axial stretching of the cells with 15 % elongation for 4 h. Actin stress fibers and nuclei were then stained and actin stress fiber angles relative to the direction of stretching were determined as in Fig. 3. The scale bar in pictures indicates 20 μm. **b** Quantification of the effect of the inhibitors on stress fiber reorganization induced by uni-axial stretch. The angles of an average of 5 actin stress fibers in a cell were counted (*n* = 50 cells for each condition). Data are expressed as the median ± interquartile. The effects of inhibitors were compared with cells not treated with inhibitors. An * indicates *p* < 0.05

The effect of RhoA/ROCK pathway signaling on actin stress fiber reorganization was then investigated using the same concentration of inhibitors as those used for analysis of JNK activation above. HBSMCs were thus pre-incubated with 5 μg/ml of the C3 exoenzyme together with 10 μg/ml lipofectamine, with 1 μM Y-27632 or with 10 μM SP600125, and were then subjected to uni-axial stretch with 15 % elongation for 4 h. Actin stress fibers were then stained with phalloidin. Typical pictures of the phalloidin-stained cells are shown in Fig. 4a*a*-*e*. The angle of the stress fibers relative to the stretch direction was then measured and a summary graph of the effects of the inhibitors is presented in Fig. 4b. Actin stress fiber reorganization was suppressed by the C3 exoenzyme (Fig. 4a*c*) and by Y-27632 (Fig. 4a*d*) but not by the JNK1 specific inhibitor SP600125 (Fig. 4a*e*.) These results indicated that the RhoA/ROCK pathway may play a significant role in actin stress fiber reorganization, although JNK1 activation had no effect on actin stress fiber reorganization induced by uni-axial stretch.

## Discussion

Mechanical overload is possibly involved in remodeling of the bladder wall, and such mechanical stress may cause morphological changes in HBSMCs. To understand the intracellular events caused by mechanical stretch, we focused on the RhoA/ROCK signaling pathway. The results demonstrated the following: 1) Uni-axial stretch activated JNK1 in HBSMCs. 2) Uni-axial stretch strongly evoked the reorganization of actin stress fibers and coordinated their orientation so that they became more perpendicular to the stretch direction. 3) The inhibitor experiments showed that the RhoA/ROCK pathway had pivotal roles in transduction of the intracellular signals induced by mechanical stretch. The stretch parameters used in this experiment were chosen to better emphasize the reaction of actin stress fiber reorganization. It should be noted that the stretch cycle used differs from the physiological condition and further assessment is needed to reflect the actual in vivo status.

RhoA mediates various extracellular signals that are induced by stimulation with agonists such as noradrenaline [17] and acetylcholine [16] in smooth muscle. RhoA was also shown to play significant roles in the signal transduction induced by mechanical stretch in endothelial cells [18] and cardiac myocytes [19]. Similarly, our data showed that RhoA appeared to have critical roles in the signaling induced by mechanical stretch in HBSMCs. However, the molecules that lead to RhoA activation by mechanical stimuli remain unclear. It is possible that scaffold proteins such as integrins or mechanosensitive ion channels [9, 20] may participate in the activation of RhoA by sensing physical stimuli via the extracellular matrix. However, further experiments are required to clarify this mechanism.

Our results suggested the existence of signal cross-linkage between RhoA/ROCK and JNK pathways in HBSMCs. Previous papers have also reported that RhoA induces JNK activation in cardiac myocytes by mechanical stretching [15]. Furthermore, stimulation of NIH 3T3 cells with an agonist such as lysophosphatidic acid (LPA) [21] or of vascular smooth muscle cells with Angiotensin II [22] causes signaling cross-talk between the ROCK pathway and JNK. There was no clear indication as to which molecules in JNK cascades are stimulated by mechanical stretch activation of RhoA/ROCK. However, some previous reports showed that ROCK activation by LPA in NIH3T3 cells induced activation of mitogen-activated protein kinase kinase 4 (MKK4), which is an upstream kinase of JNK and leads to the phosphorylation of JNK [21]. In addition, ROCK activation by activin in keratinocytes induced activation of mitogen-activated protein kinase kinase kinase 1 (MEKK1), which is an upstream kinase of MKK4, and subsequently leads to JNK activation [23]. The activation of JNK by mechanical stretch was previously shown to promote the expression of immediate early genes in vascular smooth muscle cells [6] and cardiomyocytes [7]. Although our data indicated that the JNK inhibitor SP600125 inhibited JNK activation by mechanical stretch, it should be noted that a recent paper suggested a limitation to the specificity of SP600125 and suggested the possibility that SP600125 might also inhibit other kinases (e.g., phosphatidylinositol 3-kinase) [24].

Actin stress fibers are composed of from 20 to 30 parallel actin filaments that are bundled by a bridge protein, α-actinin [10]. Actin filaments are temporally connected to bundles of polymerized type II myosin and generate tension by utilizing the actomyosin bond. The ends of actin stress fibers connect to focal adhesions where cell membranes attach to the extra-cellular matrix [10]. Under static conditions, actin stress fibers generate an isometric contraction in order to maintain a constant length with scaffolding at both ends. Cells are thought to sense extra-cellular physical signals using this isometric contraction.

In the stretch experiments of the present study, actin stress fibers were reorganized by mechanical stretch, such that stronger actin stress fibers were formed as a result of the stretch stimuli and the stress fibers were organized so that their orientation came close to being perpendicular to the stretch direction. It is unclear whether actin stress fiber angle change induces alteration of cell contractility in BSMCs. Moreover, there is little evidence that actin fiber orientation change enhances smooth muscle contractility in other types of muscle cells. Many of the cells formed projections such as filopodia in the lateral side of their cell membrane, where actin stress fibers are bound. Reorganized fibers may cause isometric tension in order to maintain the cell area. These morphological changes appear to be an adaptation response for the conservation of intracellular configurations because the formation of actin stress fibers perpendicular to the direction of stretch may restrict the movement of cell organelles. In addition, the shape of some cells was also elongated perpendicular to the direction of stretch. We also conjecture that cells may try to change their polarity by using actin stress fibers as a frame in order to reduce mechanical stress. However, there was no significant difference in the overall horizontal-vertical ratio of the cell length of stretched cells vs. unstretched cells due to their wide variety of cell shapes. The morphological changes of the stress fibers may be induced via the RhoA/ROCK pathway because the C3 exoenzyme and Y-27632 inhibited their reorganization. ROCK has been reported to participate in the induction of prominent actin stress fiber formation [11]. One of the mechanisms underlying the induction of stress fiber formation by ROCK is the phosphorylation of LIM-kinase, which is an effector of cofilin. It is considered that phosphorylated cofilin is subsequently rendered inactive and that thereby actin polymerization is ultimately stabilized [25]. Moreover, Na^+^/H^+^ exchanger 1, which is another substrate of ROCK, is activated by ROCK and subsequently induces the connection between actin filaments and cell surface proteins [26, 27]. Furthermore, mDia, which is an effector of RhoA, is reported to induce actin stress fiber reorganization together with ROCK [28]. It is reported that stress fiber orientation change due to mechanical stretch needs mDia activation in endothelial cells [18]. ROCK has also been reported to induce actinomyosin contraction via phosphorylation of at least four substrates including myosin light chain, all of which lead to increased myosin phosphorylation and increased actomyosin contractility [10].

Our results indicated that the JNK inhibitor SP600125 did not suppress actin stress fiber reorganization, therefore the JNK pathway may not participate in this reorganization. An interesting finding was reported by Kaunas et al. that a 90° change in the direction of stretch caused re-activation of JNK, when JNK activity had disappeared after the initial stretch. In addition, JNK activity was prolonged by cytochalasin D which also suppresses actin stress fiber reorganization [29]. Hence, actin cytoskeleton contraction appears to act as some sort of trigger for JNK activation. Their concept was that deformation of the actin cytoskeleton could induce biochemical signals since the stretch-induced JNK activation subsides if stress fibers are able to organize in a configuration that minimizes the perturbation by stretch.

Since the bladder wall may be subject to mechanical stress in bladder outlet obstructive disease such as benign prostatic hyperplasia, it is important to understand how cells transduce mechanical signals and adapt themselves to them. In *our in vitro* research study, activation of RhoA/ROCK pathways by mechanical stretch induced both structural and biochemical adaptations. It is possible that the consequence of actin fibers reorganization is to lead to the preservation of the cell environment by reducing mechanical stress. Since the roles of actin stress fibers in the bladder wall in vivo have been unclear to date, further investigation is required to better understand their roles.

## Conclusions

Bladder smooth muscle cells appear to have elaborate mechanisms for sensing mechanical stress and for adapting to mechanical stress overload by cytoskeletal remodeling and by activating cell growth signals such as JNK via RhoA/ROCK pathways.

## Abbreviations

HBSMCs: human bladder smooth muscle cells
JNK: c-Jun NH_2_ terminal kinase
LPA: lysophosphatidic acid
MAPK: mitogen activated protein kinase
MEKK1: mitogen-activated protein kinase kinase kinase 1
MKK4: mitogen-activated protein kinase kinase 4
PBS: phosphate buffer saline
ROCK: Rho-associated coiled-coil forming protein kinase
